# Phytoestrogens in Human Pregnancy

**DOI:** 10.1155/2012/850313

**Published:** 2012-05-14

**Authors:** John Jarrell, Warren G. Foster, David W. Kinniburgh

**Affiliations:** ^1^Department of Obstetrics and Gynecology, University of Calgary, 1403 29 NW Street, Calgary, AB, Canada T2N 2T9; ^2^Reproductive Biology Division, Department of Obstetrics and Gynecology, McMaster University, Hamilton, ON, Canada L8N 3Z5; ^3^Alberta Centre for Toxicology, University of Calgary, Calgary, AB, Canada T2N 2T9

## Abstract

*Background*. The hormonal milieu associated with pregnancy has become a focus of interest owing to potential links with the developmental origins of health and disease. Phytoestrogens are hormonally active plant-derived chemicals that may have an impact on human reproductive processes. However, developmental exposure to phytoestrogens has not been well characterized and thus our objective was to quantify phytoestrogen exposure during pregnancy and lactation. *Methods*. Women in the second trimester of pregnancy entered the study during counseling for prenatal genetic information. Women who had an indication for a genetic amniocentesis on the basis of late maternal age were approached for inclusion. They completed an environmental questionnaire; a sample of amniotic fluid was collected for karyotype, blood was collected from women during pregnancy and at birth, from the umbilical cord and breast milk. Samples were tested for the presence of daidzein and genistein by GC Mass Spectroscopy. *Findings*. Phytoestrogens are commonly found in pregnant women's serum and amniotic fluid during pregnancy. There is a sex difference in the concentrations with higher levels in amniotic fluid containing female fetuses. This difference was not present in maternal serum. Soy ingestion increases amniotic fluid phytoestrogen concentrations in female and male fetuses. The presence and concentrations of phytoestrogens did not differ in relation to common pregnancy complications or preexisting infertility.

## 1. Introduction

Phytoestrogens are naturally occurring polycyclic phenols that are isolated from certain plants [1]. They are of interest due to their weak estrogenic functions [2]. They are most commonly found in isoflavones and lignans [3]. The most common source of isoflavones is soy-based products either directly or indirectly ingested in the diet [4]. Daidzein and genistein are among the most frequently isolated isoflavones [5].

Importantly, there is considerable variation in the concentration of the compounds owing to different rates of absorption and metabolism [6]. The phytoestrogens are ingested in their beta-glycosidic state which are hydrolyzed in the intestine, absorbed and then undergo glucuronidation in the intestinal wall and the liver [6]. It is the glucuronidated compounds that circulate and are measured in the blood and urine [7, 8]. Excretion is rapid, occurring in approximately 24 hours. There have been few studies of human pregnancy kinetics, but the phytoestrogens daidzein and genistein have been found in amniotic fluid and transplacental passage of genistein has been documented in vitro in human and in vivo in rat placentae [9–12]. 

There are concerns that these chemicals may, alone or in combination with other chemicals, function as endocrine disruptors, with potentially adverse effects on male reproductive function although there are not sufficient data available to confirm these concerns [13]. One meta-analysis of fifteen placebo-controlled trials with baseline and intervention levels of testosterone, sex hormone binding globulin, free testosterone, and free androgen index failed to demonstrate that ingestion of soy foods or phytoestrogens altered testosterone bioavailability. Studies of human exposure during pregnancy have been limited in number [14]. Therefore this study was designed to evaluate the concentrations and correlations of daidzein and genistein in the amniotic fluid, maternal serum during pregnancy, at birth, the umbilical cord levels, and breast milk concentration in relation to important clinical variables and pregnancy outcomes. A parallel study from this cohort of women on the concentrations of chlorinated organic chemical exposure in pregnancy has been reported [15].

## 2. Methods

Pregnant women attending a prenatal counseling session at Foothills Hospital in Calgary, Alberta, were approached to participate in this project. Approval was obtained from the University of Calgary Ethics committee. Three hundred and twenty-three women were enrolled after their eligibility was determined. They were seeking counseling for age-related genetic counseling and all women were at least 35 years of age. The women were approached during an educational presentation for genetic testing. Although the project was very popular, the total number attending the sessions is not known.

Of those agreeing to participate, two hundred and thirty-eight completed an environmental questionnaire. Amniotic-fluid was collected for testing from three hundred and twenty-three women. Blood was collected from two hundred and nine women during the second trimester of pregnancy, one hundred and five women at the time of delivery, and ninety seven from cord samples at delivery, and forty-seven samples of breast milk were collected. The peripheral blood was collected in red topped tubes in a volume of 10–20 mL, allowed to clot at 4°C and centrifuged.

Clinical information was collected from three Calgary hospitals and three hospitals outside the Calgary Health Region. The samples were collected by the regional laboratory in specially prepared glass tubes, centrifuged and transferred to the Centre for Toxicology, University of Calgary for analysis.

Daidzein and genistein were measured by Gas Chromatography Mass Spectrometry (GSMS) using an Hewlett-Packard 6890 Gas Chromatograph equipped with a HP 5973 Mass Spectral (MS) Detector and a Hewlett-Packard Capillary Column HP-5MS (cross-linked 5% PH ME Siloxane) (30 m × 0.25 mm i.d., film thickness: 0.25 *μ*m) (Agilent Technologies, CA, USA). Breast milk was vortexed for 5 minutes before being prepared for extraction and serum and amniotic fluid were analyzed without pretreatment. Amniotic fluid was not diluted with Type I water (Milli-Q Synthesis, Millipore Ltd., ON, Canada) and was not extracted with solid phase extraction prior to liquid/liquid extraction. A 1 mL sample aliquot was spiked with internal standards and buffered with the 0.5 M acetic buffer to a pH of 5.2. Daidzein and genistein were obtained from Toronto Research Chemicals Inc., Ontario, Canada. Daidzein-d3 was obtained from Radian International, TX, USA, and Genistein-d3 was obtained from Cambridge Isotope Laboratories, Inc., MA, USA. The sample was hydrolyzed for 3 hours at 60°C with the Type H-5 Helix pomatia enzyme (Sigma-Aldrich, MO, USA). After hydrolysis, sample was diluted with Type I water (1 : 1 ratio for serum and 1 : 4 for breast milk) and sonicated for 30 minutes (Bransonic 2210, Branson Ultrasonics, CT, USA). The sample was extracted using solid phase extraction with a conditioned C18 cartridge and eluted with diethyl ether. The eluent was then dried down and reconstituted with water and then further cleaned up with hexane. The aqueous sample was then extracted with diethyl ether. The organic layer was dried with anhydrous sodium sulfate and then evaporated down to dryness under nitrogen gas at 40°C. The sample was then derivatized with N-Methyl-N-[trimethylsilyl]-trifluoroacetamide (MSTFA) (Sigma M-7891) in Dithioerythritol (Sigma D-8255), (Sigma-Aldrich, MO, USA) at 70°C for 30 minutes, and 1uL of the extract was injected on the GC/MS. The LOQ for the method was 0.5 ng/mL for both diadzein and genistein.

As the results of the concentrations were skewed, analysis was done using nonparametric statistics. Nondetectable concentrations were treated as zero. For comparison between compartments, the Wilcoxon signed ranks test was used. For correlation analysis, Spearman's test was used. Normally distributed data were compared using *t-*Test. A *P* < 0.05 was considered statistically significant.

## 3. Results

The demographics and reproductive histories of the women are summarized in Table 1. During the previous year 15.1% of the women reported that they were smokers; 8% continued to smoke during this pregnancy with an average of less than 20 cigarettes per day.

Sixty-six women reported being on a specific diet, most commonly weight reduction, and eighty-five women reported a significant weight loss of an average of 7.3 pounds. A subgroup of one hundred and six women provided detailed information on soy supplementation in their diet.

Of the pregnancies under review there were 291 births in which the following complications occurred: pregnancy-induced hypertension of 8.2%, premature labor of 3.4%, gestational diabetes of 5.2%, low birth weight of 4.8%, and Caesarian section rate of 27%. The mean birthweight was 3404 ± 533 (SD) g. There was no difference in birthweight between male and female infants. The sex ratio (male/female) was 1.13. The frequency of women reporting prior infertility was 12% in this study. Genetic analysis determined that there were two cases of Klinefelter syndrome and 2 cases of trisomy that went on to deliver.

Of the 323 women who had sampling of the amniotic fluid, 300 had testing for phytoestrogens, and of these samples, 185 (62%) contained detectable daidzein and 183 (61%) detectable genistein. The concentrations of the phytoestrogens among all samples and the concentrations in male and female pregnancies are presented in Tables 2 and 3. The serum concentrations were higher in maternal serum during pregnancy than those in amniotic fluid. There were no differences in the concentrations of daidzein and genistein in serum during pregnancy compared to serum at birth. There were significant correlations between the amniotic levels of daidzein with both daidzein and genistein and maternal serum during pregnancy and at birth and cord serum with the exception of serum at birth genistein levels. There were significant correlations of amniotic fluid genistein with both daidzein and genistein in all compartments (Table 4). The degree of correlation was greater for genistein than that for daidzein. Breast milk samples of daidzein correlated with amniotic fluid genistein while breast milk samples of genistein correlated with amniotic fluid daidzein levels (Table 4).

Notably there were significantly higher concentrations in the amniotic fluid of pregnancies with female fetuses compared to amniotic fluid of pregnancies with male pregnancies. Amniotic fluid daidzein concentrations from male pregnancies were 48% of the concentrations from female pregnancies while the amniotic fluid genistein concentrations from male pregnancies were 54% of the concentrations from female pregnancies *P* < 0.05. There were no sex-related differences in serum during pregnancy, at birth, cord serum, or breast milk. Although the mean concentrations of cord daidzein and genistein were higher than maternal blood at birth, these differences were not statistically significant.

**Figure 1:**
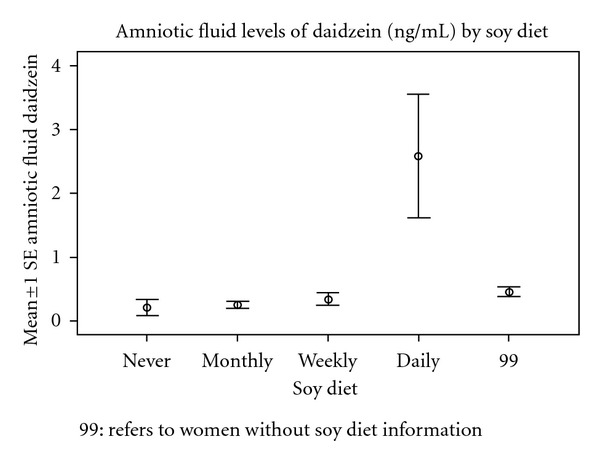
The amniotic fluid levels of Daidzein (ng/mL) by soy diet.

**Figure 2:**
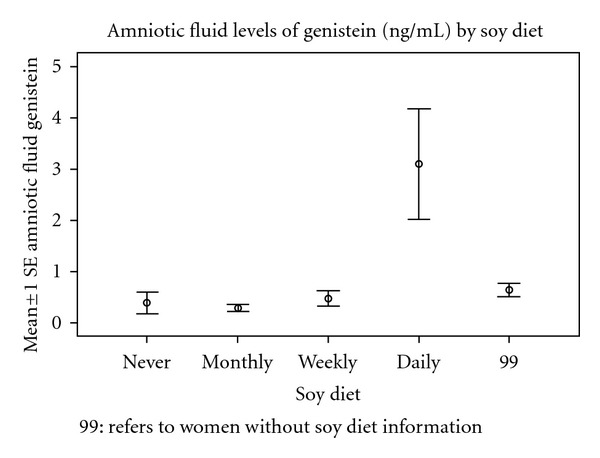
The amniotic fluid levels of Genistein (ng/mL) by soy diet.

**Figure 3:**
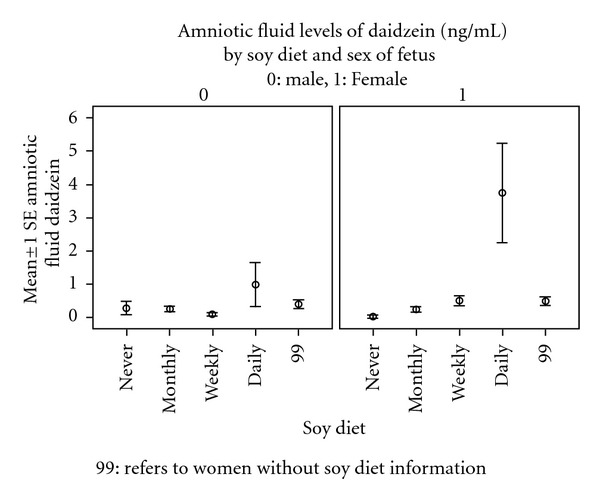
The amniotic fluid levels of Daidzein (ng/mL) by soy diet and sex of fetus.

**Figure 4:**
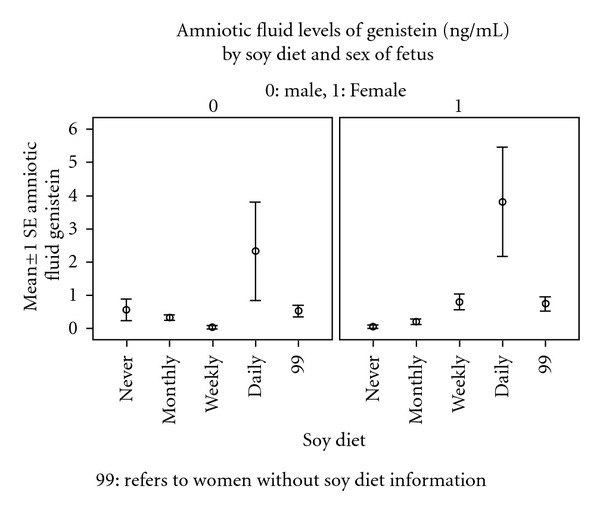
The amniotic fluid levels of Genistein (ng/mL) by soy diet and sex of fetus.

Among women reporting their soy intake, there was an increase in the amniotic fluid levels of daidzein and genistein (Figures 1 and 2). There was no difference in the frequencies of intake between male and female fetuses (Table 5). However, women who reported the use of soy products were found to have higher concentrations of daidzein and genistein in the amniotic fluid in pregnancies with a female fetus than with a male fetus (Figures 3 and 4).

The concentrations of daidzein and genistein did not differ when the levels were compared among women with complications of pregnancy including premature labor, pregnancy-induced hypertension, gestational diabetes, low birth weight, or Caesarian section. There were no differences in the amniotic fluid levels of phytoestrogens among women reporting prior infertility compared to those who did not report infertility. There was no increase in the rates of infertility among women who ingested higher concentrations of soy products.

## 4. Discussion

The results of these investigations indicate that the phytoestrogens are commonly identified in the amniotic fluid and blood of women and fetuses during pregnancy as previously reported [16]. The concentrations are higher in the maternal serum than those in the amniotic fluid although there were no significant differences between amniotic fluid and serum at birth, cord serum, or breast milk. The significant correlations indicate that measures of cord blood phytoestrogens can provide a reasonable estimate of the levels that were present in amniotic fluid, earlier in pregnancy.

One significant finding not previously reported was the difference noted between male and female fetuses with significantly higher concentrations among the female fetuses. No immediate explanation for this is apparent. The birthweights of the male and female infants were similar suggesting that fetal size would not be an explanation. The sex differences in amniotic fluid daidzein and genistein suggest that there may be a differential metabolic handling of these chemicals during fetal life. Previous studies of adult human urine levels of phytoestrogens have not demonstrated sex-related differences however [17]. There were differences in intestinal metabolism of microflora noted in other human studies in response to soy-augmented diet [18].

Soy levels of ingestion were assessed in 102/231 women, and among these women, there was a higher level of daidzein and genistein in the amniotic fluid. This did not appear to be the explanation for sex differences as the frequency of the soy ingestion patterns did not differ by sex. The data suggest that dietary soy concentrations should be considered in the analysis of amniotic fluid concentrations of phytoestrogens.

This finding of a sex difference should be confirmed, as early exposure to these chemicals has been suggested as an important area of research due to the possibility that these chemicals can act alone or in combination with other chemicals as endocrine disruptors [19]. Safety of these chemicals in relation to androgen metabolism in males has been reported [20].

There was no difference in the concentrations between the fetus and mother at birth, consistent with previous studies of the transplacental passage of the chemicals [9, 21].

The strengths of the study are based on it being a prospective study of a large sample size of pregnant women employing direct measurements of exposure with state-of-the-art techniques. The study provides information that demonstrates a dose relationship of phytoestrogens to soy consumption.

The limitations are based on the sample size being too small to evaluate exposure with specific adverse health outcomes, and the study population is not representative of the general population of pregnant women. Also, the samples of cord blood and breast milk are available for only a small segment of the population. In relation to the soy ingestion, there are no data to indicate the type of soy or the last time of ingestion.

In summary, we have demonstrated that pregnant women of advanced maternal age have exposure to phytoestrogens which can be measured in the amniotic fluid and umbilical cord blood demonstrating fetal exposure. Moreover, when soy consumption was considered, there was a dose response relationship for phytoestrogens in the amniotic fluid. Finally, concentrations of daidzein were significantly higher in the amniotic fluid of female versus male fetuses suggesting potential sex differences in metabolic capacity of the fetus.

## Figures and Tables

**Table 1:** Clinical variables.

	*N*	Minimum	Maximum	Mean	Standard deviation
Age	238	35	48	39.03	2.29
BMI	238	16.47	42.77	24.08	7.11
Gravida	319	1.00	10	2.78	1.52
Parity	317	0	6	0.94	0.93
Term	316	0	6	0.89	0.94
Preterm	316	0	1	0.04	0.21
Stillbirth	318	0	1	0.00	0.06
Spont Ab	316	0	5	0.54	0.89
Therapy Ab	315	0	3	0.30	0.60
Smokers	238	0	1	0.08	0.27
Number of Cigarettes	238	0	20	0.60	2.52

Spont Ab: Spontaneous abortion.

**Table 2:** Phytoestrogen concentration over the course of pregnancy.

	Phytoestrogen concentrations in human pregnancy			
	*N*	Mean	Standard error	Standard deviation
Amniotic fluid daidzein	300	0.60	0.08	1.41
Amniotic fluid genistein	300	0.77	0.10	1.78
Serum during pregnancy daidzein	215	2.88	0.88	12.96
Serum during pregnancy genistein	215	5.18	1.84	27.02
Serum at birth daidzein	97	1.21	0.55	5.45
Serum at birth genistein	100	1.48	0.84	8.40
Cord serum daidzein	75	2.30	0.81	7.02
Cord serum genistein	85	2.68	1.07	9.87
Breast milk daidzein	90	0.25	0.11	1.03
Breast milk genistein	84	0.61	0.24	2.20

**Table 3:** Phytoestrogen concentration during pregnancy by sex of fetus.

Phytoestrogen concentration during pregnancy by sex of fetus					
	Sex	*N*	Mean	Standard deviation	Standard error mean
Amniotic fluid daidzein	Male	152	0.41	0.82	0.07
	Female	136	0.85	1.88	0.16
Amniotic fluid genistein	Male	152	0.55	1.25	0.10
	Female	136	1.04	2.27	0.19
Serum during pregnancy daidzein	Male	118	2.77	13.36	1.23
	Female	94	3.08	12.71	1.31
Serum during Pregnancy genistein	Male	118	5.16	27.23	2.51
	Female	94	5.36	27.33	2.82
Serum at birth daidzein	Male	55	0.75	1.69	0.23
	Female	42	1.82	8.07	1.25
Serum at birth genistein	Male	56	0.68	1.56	0.21
	Female	43	2.55	12.69	1.94
Cord serum daidzein	Male	37	2.66	8.28	1.36
	Female	36	2.02	5.77	0.96
Cord serum genistein	Male	45	3.27	11.64	1.73
	Female	38	2.08	7.70	1.25
Breast milk daidzein	Male	43	0.36	1.41	0.21
	Female	46	0.16	0.49	0.07
Breast milk genistein	Male	42	0.87	2.86	0.44
	Female	42	0.36	1.21	0.19

**Table 4:** Correlation analysis of daidzein and genistein between the tissue compartments of amniotic fluid, maternal serum during pregnancy and at birth, cord serum, and breast milk.

		Amniotic fluid daidzein	Amniotic fluid genistein	Serum in pregnancy daidzein	Serum in pregnancy genistein	Serum at birth daidzein	Serum at birth genistein	Cord serum daidzein	Cord serum genistein	Breast milk daidzein	Breast milk genistein
Amniotic fluid daidzein	Rho	1.000	0.784**	0.244**	0.204**	0.261*	0.165	0.263*	0.234*	0.210	0.265*
	*P*		0.000	0.000	0.003	0.011	0.106	0.028	0.034	0.053	0.018
	*N*	300.000	300.000	206.000	206.000	94.000	97.000	70.000	82.000	86.000	80.000

Amniotic fluid genistein	Rho	0.784**	1.000	0.233**	0.278**	0.352**	0.322**	0.351**	0.289*	0.226*	0.208
	*P*	0.000		0.001	0.000	0.000	0.001	0.003	0.009	0.036	0.064
	*N*	300.000	300.000	206.000	206.000	94.000	97.000	70.000	82.000	86.000	80.000

Serum in pregnancy daidzein	Rho	0.244**	0.233**	1.000	0.853**	0.299**	0.257*	0.352**	0.304**	0.269*	0.343**
	*P*	0.000	0.001		0.000	0.003	0.010	0.003	0.005	0.015	0.002
	*N*	206.000	206.000	215.000	215.000	95.000	98.000	71.000	82.000	81.000	77.000

Serum in pregnancy genistein	Rho	0.204**	0.278**	0.853**	1.000	0.287**	0.267**	0.313**	0.288**	0.278*	0.373**
	*P*	0.003	0.000	0.000		0.005	0.008	0.008	0.009	0.012	0.001
	*N*	206.000	206.000	215.000	215.000	95.000	98.000	71.000	82.000	81.000	77.000

Serum at birth daidzein	Rho	0.261*	0.352**	0.299**	0.287**	1.000	0.885**	0.824**	0.634**	0.168	0.078
	*P*	0.011	0.000	0.003	0.005		0.000	0.000	0.000	0.197	0.562
	*N*	94.000	94.000	95.000	95.000	97.000	94.000	64.000	74.000	61.000	57.000

Serum at birth genistein	Rho	0.165	0.322**	0.257*	0.267**	0.885**	1.000	0.704**	0.646**	0.100	0.031
	*P*	0.106	0.001	0.010	0.008	0.000		0.000	0.000	0.446	0.824
	*N*	97.000	97.000	98.000	98.000	94.000	100.000	66.000	77.000	60.000	55.000

Cord serum daidzein	Rho	0.263*	0.351**	0.352**	0.313**	0.824**	0.704**	1.000	0.865**	0.452**	0.233
	*P*	0.028	0.003	0.003	0.008	0.000	0.000		0.000	0.002	0.149
	*N*	70.000	70.000	71.000	71.000	64.000	66.000	75.000	72.000	44.000	40.000

Cord serum genistein	Rho	0.234*	0.289**	0.304**	0.288**	0.634**	0.646**	0.865**	1.000	0.540**	0.427**
	*P*	0.034	0.009	0.005	0.009	0.000	0.000	0.000		0.000	0.004
	*N*	82.000	82.000	82.000	82.000	74.000	77.000	72.000	85.000	49.000	44.000

Breast milk daidzein	Rho	0.210	0.226*	0.269*	0.278*	0.168	0.100	0.452**	0.540**	1.000	0.794**
	*P*	0.053	0.036	0.015	0.012	0.197	0.446	0.002	0.000		0.000
	*N*	86.000	86.000	81.000	81.000	61.000	60.000	44.000	49.000	90.000	82.000

Breast milk genistein	Rho	0.265*	0.208	0.343**	0.373**	0.078	0.031	0.233	0.427**	0.794**	1.000
	*P*	0.018	0.064	0.002	0.001	0.562	0.824	0.149	0.004	0.000	
	*N*	80.000	80.000	77.000	77.000	57.000	55.000	40.000	44.000	82.000	84.000

**Correlation is significant at the 0.01 level (2-tailed).

*Correlation is significant at the 0.05 level (2-tailed).

**Table 5:** The relationship of soy in the diet to the sex of the fetus.

	Frequency	Male	Female	Total
Soy in diet	never	12	7	19
	monthly	28	20	48
	weekly	10	11	21
	daily	5	9	14
	No response	69	60	129

	Total	124	107	231
